# Exogenous glucagon-like peptide-1 attenuates the glycaemic response to postpyloric nutrient infusion in critically ill patients with type-2 diabetes

**DOI:** 10.1186/cc9983

**Published:** 2011-01-21

**Authors:** Adam M Deane, Matthew J Summers, Antony V Zaknic, Marianne J Chapman, Robert JL Fraser, Anna E Di Bartolomeo, Judith M Wishart, Michael Horowitz

**Affiliations:** 1 Discipline of Acute Care Medicine, University of Adelaide, North Terrace, Adelaide, South Australia, 5000, Australia; 2 Intensive Care Unit, Level 4, Emergency Services Building, Royal Adelaide Hospital, North Terrace, Adelaide, South Australia, 5000, Australia; 3 National Health and Medical Research Council of Australia Centre for Clinical Research Excellence in Nutritional Physiology and Outcomes, Level 6, Eleanor Harrald Building, North Terrace, Adelaide, South Australia, 5000, Australia; 4 Discipline of Medicine, University of Adelaide, Royal Adelaide Hospital, Level 6 Eleanor Harrald Building, North Terrace, Adelaide, South Australia, 5000, Australia; 5 Investigation and Procedures Unit, Repatriation General Hospital, Daws Road, Daw Park, South Australia, 5041, Australia

## Abstract

**Introduction:**

Glucagon-like peptide-1 (GLP-1) attenuates the glycaemic response to small intestinal nutrient infusion in stress-induced hyperglycaemia and reduces fasting glucose concentrations in critically ill patients with type-2 diabetes. The objective of this study was to evaluate the effects of acute administration of GLP-1 on the glycaemic response to small intestinal nutrient infusion in critically ill patients with pre-existing type-2 diabetes.

**Methods:**

Eleven critically ill mechanically-ventilated patients with known type-2 diabetes received intravenous infusions of GLP-1 (1.2 pmol/kg/minute) and placebo from t = 0 to 270 minutes on separate days in randomised double-blind fashion. Between t = 30 to 270 minutes a liquid nutrient was infused intraduodenally at a rate of 1 kcal/min via a naso-enteric catheter. Blood glucose, serum insulin and C-peptide, and plasma glucagon were measured. Data are mean ± SEM.

**Results:**

GLP-1 attenuated the overall glycaemic response to nutrient (blood glucose AUC_30-270 min_: GLP-1 2,244 ± 184 vs. placebo 2,679 ± 233 mmol/l/minute; *P *= 0.02). Blood glucose was maintained at < 10 mmol/l in 6/11 patients when receiving GLP-1 and 4/11 with placebo. GLP-1 increased serum insulin at 270 minutes (GLP-1: 23.4 ± 6.7 vs. placebo: 16.4 ± 5.5 mU/l; *P *< 0.05), but had no effect on the change in plasma glucagon.

**Conclusions:**

Exogenous GLP-1 in a dose of 1.2 pmol/kg/minute attenuates the glycaemic response to small intestinal nutrient in critically ill patients with type-2 diabetes. Given the modest magnitude of the reduction in glycaemia the effects of GLP-1 at higher doses and/or when administered in combination with insulin, warrant evaluation in this group.

**Trial registration:**

ANZCTR:ACTRN12610000185066

## Introduction

The management of hyperglycaemia in the critically ill is an important, and contentious, issue [1,2]. In critically ill patients the ideal glycaemic range is uncertain, but is likely to be ≤ 10 mmol/l [1]. When compared to critically ill patients with so-called 'stress hyperglycaemia' those with known diabetes are at greater risk of complications from hypoglycaemia, yet appear to be less vulnerable to the toxicity of hyperglycaemia [2]. The mechanisms underlying hyperglycaemia in critically ill patients with known diabetes are complex, but include relative insulin insufficiency, insulin resistance and hyperglucagonaemia [3].

Glucagon-like peptide-1 (GLP-1), secreted from enteroendocrine L-cells in response to intestinal nutrient, has the capacity to lower blood glucose [4]. In ambulant type-2 diabetics, exogenous GLP-1 decreases blood glucose via stimulation of insulin and suppression of glucagon secretion, as well as slowing of gastric emptying [5]. As the effects of GLP-1 on insulin and glucagon are glucose-dependent the risk of hypoglycaemia with its administration is low [6]. In ambulant type-2 diabetics the GLP-1 analogue, exenatide, has been reported to achieve comparable reductions in glycated haemoglobin, but with less hypoglycaemia and a reduction in glycaemic variability when compared to insulin glargine [7]. For the above reasons GLP-1 is a potentially attractive therapeutic option for the management of hyperglycaemia in the substantial number of critically ill patients with pre-existing type-2 diabetes. This concept has been strengthened by our recent reports that acute administration of GLP-1 markedly attenuates the glycaemic response to enteral nutrients in critically ill patients with stress-hyperglycaemia [8,9].

The primary aim of this study was to evaluate the effects of an acute, exogenous GLP-1 infusion (1.2 pmol/kg/minute) on the glycaemic response to a postpyloric nutrient infusion in critically ill patients with known type-2 diabetes. Secondary aims were to explore mechanism(s) underlying glucose-lowering if demonstrated, and to determine whether glycaemic excursions could be limited to < 10 mmol/l with GLP-1 administration.

## Materials and methods

### Subjects

Critically ill adult patients known to have pre-existing type-2 diabetes that were admitted to the Royal Adelaide Hospital Intensive Care Unit between Jan 2009 and May 2010 were studied. Patients were included if aged greater than 17 years and likely to remain mechanically ventilated for > 48 hours. Exclusion criteria were pregnancy, contraindication to enteral feeding or post-pyloric catheter insertion, acute pancreatitis and previous surgery on the oesophagus, stomach or duodenum.

Subject demographic data are presented in Table 1. In 6 of the 11 subjects their diabetes was managed by diet alone. Glycated haemoglobin ranged from 6.0 to 12.2% and the body mass index (BMI) ranged from 20.2 to 50.2 kg/m^2^. Admission diagnoses were categorised as sepsis (*n *= 5), trauma (3), cardiac (2) and respiratory (1). Nine patients had received exogenous insulin during their admission prior to enrolment. The study was approved by the Human Ethics Committee of the Royal Adelaide Hospital and performed according to local requirements for the conduct of research on unconscious patients. Written, informed consent was obtained from the next of kin.

**Table 1:** Patient demographics, mean ± SEM

**Age (years)**	59 ± 5
**Gender (Male : Female)**	9 : 2
**Body mass index (kg/m^2^)**	33 ± 3
**Glycated haemoglobin (%)**	8.5 ± 0.6
**Anti-diabetic treatment prior to hospital admission (n)**	Metformin (2)
	Sulfonyurea (2)
	Insulin (1)
	Dietary regimen (6)
**Admission diagnosis (n)**	**Sepsis (5)**
	Pneumonia (2)
	Pyelonephritis
	Influenza A (H1N1) virus
	Septic shock from unknown focus
	**Trauma (3)**
	Isolated Chest (2)
	Multi-trauma
	**Cardiac failure and/or cardiogenic shock (2)**
	Cardiogenic Pulmonary Oedema
	Cardiogenic shock
	**Exacerbation of Chronic obstructive pulmonary disease (1)**
**APACHE II Score**	21 ± 3
Admission	19 ± 3
First study day	6 ± 1
**Days in ICU prior to first study day**	Exogenous catecholamines (3)
**Medications (n)**	Exogenous steroids (2)
	Exogenous catecholamines and steroids (2)
	
Acute renal impairment (n) *	6
Acute hepatic impairment (n)**	2

* Acute renal impairment defined as serum creatinine > 150 umol/l, or rise in creatinine > 80 umol/l, or patient receiving renal replacement therapy when not on chronic dialysis on first day of study.

** Acute hepatic impairment defined as patients with serum bilirubin > 24 umol/l, and alanine aminotransferase (ALT) > 55 U/l, and international normalised ratio (INR) ≥ 1.3 on first day of study.

APACHE II, Acute Physiology and Chronic Health Evaluation II.

### Study protocol

**Figure 1:**
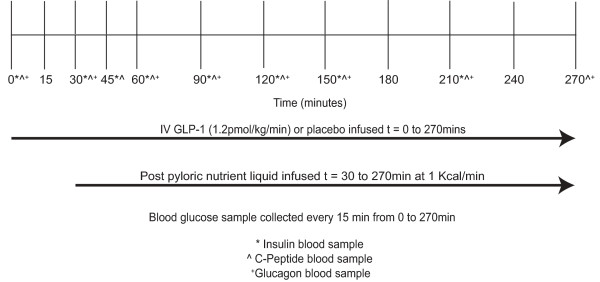
**Time line**. A randomised, double-blind, placebo-controlled, cross-over study with study drug infused for 30 minutes prior to administration of small intestinal nutrient infusion.

The protocol is summarised in Figure 1. Patients were studied over two consecutive days, in which they received intravenous (IV) GLP-1 or placebo in a randomised, double-blind fashion, as described previously [8]. In brief, a postpyloric feeding catheter was inserted using an electromagnetic technique [10]. Enteral feeding was ceased at least six hours and IV insulin ceased a minimum of two hours before the commencement of the study drug. Synthetic GLP-1-(7-36) amide acetate (Bachem, Bubendorf, Germany) was reconstituted by the Royal Adelaide Hospital Department of Pharmacy, as a solution in 4% albumin and allocation concealment was maintained throughout. Both GLP-1 (1.2 pmol/kg/minute) and control (4% albumin) were infused at a rate of 1 ml/minute for 270 minutes [8]. At t = 30 minutes a mixed nutrient liquid, Ensure^® ^(Abbott, Victoria, Australia), was delivered into the small intestine continuously at a rate of 1.0 ml/minute for four hours (that is, at 1 kcal/minute between t = 30 to 270 minutes). Arterial blood samples were obtained immediately prior to starting the IV (t = 0 minutes) and intraduodenal (t = 30 minutes) infusions and then at 15 minute intervals for measurement of blood glucose [8]. Blood samples were also collected at timed intervals for measurements of serum insulin and C-peptide, as well as plasma glucagon. If the recorded blood glucose was > 15 mmol/l the IV infusion was ceased, insulin administered, and the study terminated at that time.

### Data analysis

Blood glucose was measured at the bedside using a portable glucometer [8]. Blood was collected for serum and plasma as described previously [8]. Insulin was measured by enzyme-linked immunosorbent assay (ELISA) (EZHI-14K, Millpore, Billerica, MA, USA). The sensitivity of the assay was 0.2 mU/L and the coefficient of variation was 6% within, and 10.3% between, assays. Serum C-peptide was measured by ELISA (Immulite 2000 C-peptide, Siemens Healthcare Diagnostics, Deerfield, IL, USA) and the lower and upper analytical limits were 33 pmol/l and 6,620 pmol/l respectively. The intraassay coefficient of variation was 4.8%. Plasma glucagon was measured by radioimmunoassay (GL-32K, Millipore). The minimum detectable limit was 20 pg/ml and maximum limit was 200 pg/ml, and the intra- and inter-assay coefficients of variations were 3.9% and 5.5% respectively [11]. Free Fatty Acids were measured by spectrophotometric determination using a Randox NEFA kit (FA115, Randox Laboratories, Crumlin, County Antrim, UK). The sensitivity of the assay was 0.1 mmol/L and the inter-assay co-efficient of variation was 4.7%.

### Statistical analysis

Data are presented as mean ± SEM. Areas under curve (AUC) were calculated using the trapezoidal rule. Power calculations were performed using previous data [8] - complete data were required in 10 subjects to detect an absolute difference in the glycaemic response to nutrient (that is, AUC_30-270 min_) of 345 mmol/l/minute at a two-sided alpha level of 0.05 with 80% power. In cases in which the study was terminated because the blood glucose was > 15 mmol/l the last glucose measurement was used for all subsequent measurements, that is, 'last observation carried forward' [12]. There was a trend for baseline plasma glucagon concentrations to vary between study days and, accordingly, glucagon is also presented as Δ from the commencement of study drug (that is, t = 0). Depending on normality the differences between intervention and placebo were assessed using Student's paired *t*-test. Data were evaluated for potential carry over effects. In addition to summary measurements (AUC), individual time points at baseline (t = 0 minutes), prior to commencing feed (t = 30 minutes) and study end (t = 270 minutes) were chosen *a priori *for analysis [8]. The relationships between the magnitude of the change in blood glucose with glycated haemoglobin, Acute Physiology and Chronic Health Evaluation (APACHE) II score, and baseline glucose were evaluated using linear regression [13]. The null hypothesis was rejected at the 0.05 significance. Statistical analyses were performed using SPSS (Version 16.0, IBM, St Leonards NSW, Australia).

## Results

Adverse gastrointestinal effects, such as nausea and/or vomiting, were not evident during the study. The study was terminated prematurely in three patients during placebo (patients number 5, 6 and 10 at 90, 120 and 150 minutes, respectively) and one patient receiving GLP-1 (patient 10 at 165 minutes) as blood glucose reached the predetermined cut-off (> 15 mmol/l).

### Blood glucose

**Figure 2:**
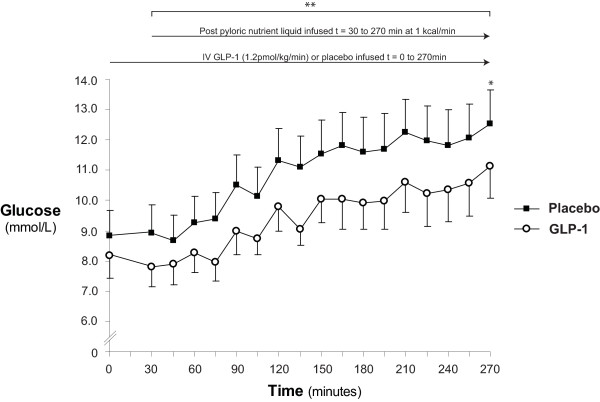
**Blood glucose**. When compared to placebo glucagon-like peptide-1 (GLP-1) caused a reduction in blood glucose at the end of the study (* at t = 270 minutes: GLP-1: 11.1 ± 1.1 vs. placebo: 12.6 ± 1.2; *P *= 0.02) and ameliorated glycaemia throughout the entire postpyloric nutrient infusion (** AUC_30 to 270 minute_: GLP-1 2,244 ± 184 vs. placebo 2,679 ± 233 mmol/l/minute; *P *= 0.02).

Blood glucose concentrations are shown in Figure 2. At the commencement of the intravenous infusion (t = 0 minutes) there was no difference in blood glucose (GLP-1 8.2 ± 0.7 vs. placebo 8.8 ± 0.9 mmol/l; *P *= 0.40). Similarly, at the end of the 'fasting' period (t = 30 minutes) GLP-1 had no significant effect on blood glucose (GLP-1 7.8 ± 0.6 vs. placebo 8.9 ± 0.9 mmol/l; *P *= 0.17). In response to nutrient infusion blood glucose increased on both days (Δ glucose = 270 minutes - 0 minutes; P < 0.01 for both). GLP-1 reduced the peak glycaemic excursion (GLP-1: 11.4 ± 0.9 vs. placebo 12.7 ± 1.1 mmol/l; *P *= 0.04) and overall glycaemic response to nutrient (AUC_30-270 minutes_: GLP-1: 2,244 ± 184 vs. placebo: 2,679 ± 233 mmol/l/minute; *P *= 0.02). During the small intestinal nutrient infusion glycaemia was maintained at < 10 mmol/l in 6/11 patients receiving GLP-1 and 4/11 patients during placebo. At study end there was a reduction in glycaemia during GLP-1 (at t = 270 minutes: GLP-1: 11.1 ± 1.1 vs. placebo: 12.6 ± 1.2 mmol/l; *P *= 0.02).

### Serum insulin

**Figure 3:**
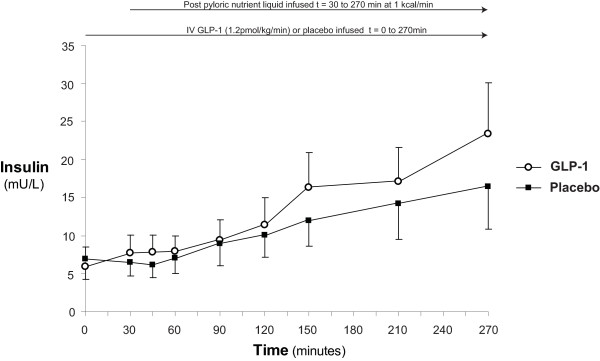
**Serum insulin**. When compared to placebo glucagon-like peptide-1 (GLP-1) caused an insulinotropic response (*** at t = 270 minutes: GLP-1: 23.4 ± 6.7 vs. placebo: 16.4 ± 5.5 mU/l; *P *< 0.05).

Serum insulin concentrations are shown in Figure 3. During GLP-1 infusion an insulinotropic effect was evident (t = 0 minutes: 5.9 ± 1.7 mU/l vs. t = 270 minutes: 23.4 ± 6.7 mU/l; *P *= 0.02), while there was only a trend for increased serum insulin during placebo (t = 0 minutes: 7.0 ± 1.5 vs. t = 270 minutes: 16.4 ± 5.5; *P *= 0.10). At the commencement of the IV infusion and at the end of the 'fasting' period GLP-1 had no effect on insulin (at t = 0 minutes: GLP-1 5.9 ± 1.7 vs. placebo 7.0 ± 1.5 mU/l; *P *= 0.30, and at t = 30 minutes: GLP-1: 7.7 ± 2.4 vs. placebo: 6.4 ± 1.7 mU/l; *P *= 0.35). However, at study end there was an increase in serum insulin during GLP-1 when compared to placebo (at t = 270 minutes: GLP-1: 23.4 ± 6.7 vs. placebo: 16.4 ± 5.5 mU/l; *P *< 0.05). There was no difference in the insulin AUC_0-270 minutes _(GLP-1: 3,076 ± 927 vs. placebo: 2,699 ± 787 mU/l/minute; *P *= 0.45).

### Serum C-peptide

**Figure 4:**
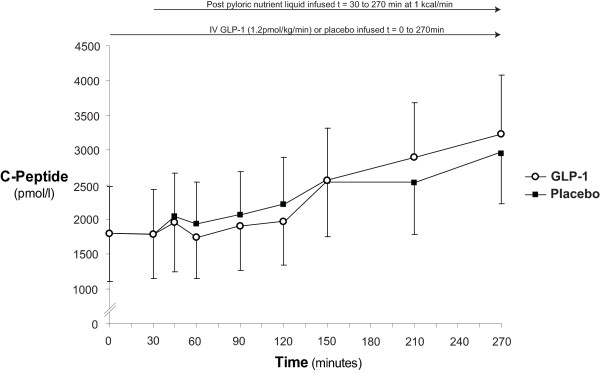
**Serum C-peptide**. When compared to placebo glucagon-like peptide-1 (GLP-1) caused no effect on C-peptide concentrations.

Serum C-peptide was greater than the maximum limit in one patient during GLP-1 infusion and was recorded as 6,620 pmol/l. Mean C-peptide concentrations are shown in Figure 4. In response to nutrient infusion there was an increment in C-peptide on both study days ((GLP-1 at t = 0 minutes 1,789 ± 689 vs. t = 270 minutes 3,227 ± 851 pmol/l.min; *P *= 0.02) and (placebo at t = 0 minutes 1,793 ± 567 vs. 2,950 ± 845 pmol/l/minute; *P *= 0.03)). At the predefined time-points, GLP-1 had no effect on serum C-peptide (at t = 0; GLP: 1,789 ± 689 vs. placebo: 1,793 ± 567 pmol/l *P *= 0.98, at t = 30; GLP: 1,786 ± 642 vs. placebo: 1,779 ± 556 pmol/l; *P *= 0.97, and at t = 270 GLP-1: 3,227 ± 851 vs. placebo: 2,950 ± 845 pmol/l; *P *= 0.38) and there was no affect on AUC_0-270 minutes _(GLP: 6.29 vs. placebo 6.31 mmol/l/minute; *P *= 0.97)

### Plasma glucagon

**Figure 5:**
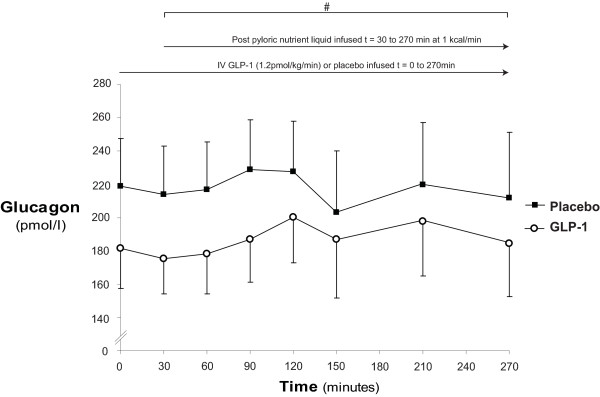
**Plasma glucagon**. When compared to placebo glucagon-like peptide-1 (GLP-1) caused a reduction in glucagon concentration AUC (# *P *< 0.01) and strong trend to decreased glucagon concentrations at baseline, commencement of feeding and completion of study (*P *= 0.06, *P *= 0.06 and *P *= 0.11 respectively).

**Figure 6:**
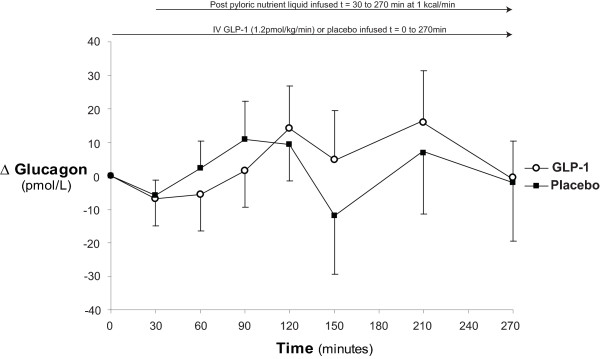
**Change in plasma glucagon**. When compared to placebo glucagon-like peptide-1 (GLP-1) caused no apparent effect on change in plasma glucagon concentrations from baseline.

Plasma glucagon concentrations are shown in Figure 5. These were greater than the maximum detectable limit throughout the study period during placebo in one patient. Postprandial suppression of glucagon was not observed during GLP-1 or placebo. There was a strong trend for lower glucagon concentrations on the day of GLP-1 administration, including baseline (at t = 0 minutes: GLP-1: 181 ± 24 vs. placebo: 219 ± 29 pmol/l; *P *= 0.06, at t = 30 minutes: GLP-1: 175 ± 21 vs. placebo: 214 ± 29: *P *= 0.06, and at t = 270 minutes: GLP-1: 184 ± 32 vs. placebo: 212 ± 39; *P *= 0.11) so that plasma glucagon was lower on the day of GLP-1 (AUC_0-270 minutes_; *P *< 0.01). However, when data were evaluated as changes from fasting concentration (Δ_glucagon_) GLP-1 had no effect Δ_glucagon _(t = 30: GLP-1: -6.9 ± 8.2 vs. placebo: -5.8 ± 4.5; *P *= 0.89, and t = 270: GLP-1: -0.6 ± 10.9 vs. placebo -2.075 ± 17.4; *P *= 0.94). (Figure 6)

### Serum non-esterified fatty acids

**Figure 7:**
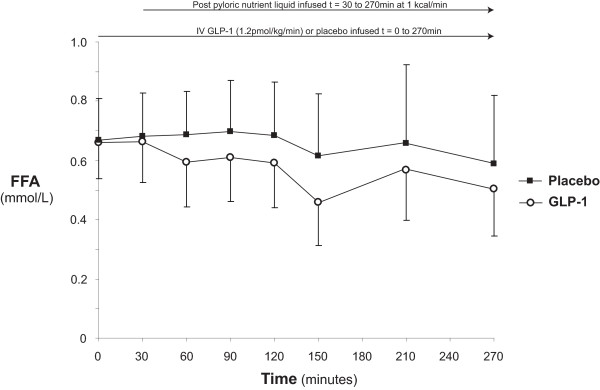
**Serum non-esterified fatty acids**. When compared to placebo glucagon-like peptide-1 (GLP-1) caused comparable effects on NEFA.

Serum non-esterified fatty acid (NEFA) concentrations are shown in Figure 7. Fasting NEFA concentrations were similar on both days (at t = 0 minutes: GLP-1: 0.66 ± 0.12 vs. placebo: 0.67 ± 0.14 mmol/l; *P *= 0.93). The nutrient infusion had no effect on NEFA. GLP-1 did not have a detectable effect on fatty acids (at t = 30 minutes: GLP-1: 0.66 ± 0.14 vs. placebo: 0.68 ± 0.14 mmol/l; *P *= 0.82, at t = 270 minutes: GLP-1: 0.51 ± 0.19 vs. placebo: 0.59 ± 0.18; *P *= 0.44, and AUC_0-270 minutes_: GLP-1: 166 ± 40 vs. placebo: 187 ± 48 mmol/l/minute; *P *= 0.21)

### Relationships to glucose-lowering

When the glycaemic response to nutrient infusion was greater, the magnitude of lowering that was observed during GLP-1 IV infusion was also increased (*r*^*2 *^= 0.38; *P *< 0.05) (that is, glucose-lowering was apparently dependent on the blood glucose). There was a trend for an association between the magnitude of glucose lowering and the APACHE II on the first study day (*r*^*2 *^= 0.31; *P *= 0.07). There was no association between glucose-lowering and glycated haemoglobin or body mass index (data not shown).

## Discussion

Our major observation is that an acute exogenous administration of GLP-1 (1.2 pmol/kg/minute) attenuates the glycaemic response to small intestinal nutrient infusion in critically ill patients with known type-2 diabetes. This effect is attributable, at least in part, to relative insulin stimulation. While the study establishes that GLP-1 has the capacity to reduce glycaemia in this group, during GLP-1 infusion glycaemic excursions were limited to < 10 mmol/l in approximately 50% of patients. There was evidence that the glucose-lowering effect of GLP-1 was glucose-dependent (that is, the greater the glucose concentrations during placebo, the greater the reduction in glucose during GLP-1). Small intestinal nutrient did not suppress glucagon in critically ill patients with type-2 diabetes during either placebo or GLP-1 infusion.

The dose of GLP-1 was selected based on previous studies [8,9,13,14]. In ambulant type-2 diabetics, GLP-1 at higher doses (2.4 pmol/kg/minute) has a greater glucose-lowering effect, but is also associated with increased adverse effects, particularly nausea and vomiting [15]. Such adverse effects may, potentially, be less common in sedated patient receiving small intestinal feeding, as opposed to nutrient administered orally to alert subjects. In view of our observations the effects of GLP-1 (or its analogues) at greater doses and/or in combination with insulin merit evaluation [16,17]. The feeding regimen was also based on our previous study in which nutrient was administered via a postpyloric tube [8]. Slowing of gastric emptying contributes to the glucose-lowering effect of exogenous GLP-1 in health, type-2 diabetics, and critically ill patients following an intragastric 'meal' [9,18,19]. Accordingly, the magnitude of the reduction in blood glucose is anticipated to be greater during intragastric feeding, particularly in those patients in whom gastric emptying is relatively normal. The rate of small intestinal nutrient infusion (1 kcal/minute) is less than optimal for maintaining nutritional requirement in this group. However, based our previous observations in non-diabetics [8], administering more calories increased the likelihood of unacceptable hyperglycaemia during placebo. While gastric emptying is frequently delayed in the critically ill, often markedly, [20] and the rate of gastric emptying of nutrients in this group may approximate 1 kcal/minute, in health the rate is usually 1 to 4 kcal/minute [9]. As the relationship between glycaemia and the rate of carbohydrate entry into the small intestine is non-linear in health [21], it is likely that a small intestinal feeding rate or gastric emptying > 1 kcal/minute will lead to greater glycaemic excursions than observed in the current study. Other limitations of this study should be recognised. No reduction in fasting glycaemia was observed, probably reflecting the short duration of fasting and GLP-1 infusion (30 minutes) and the small cohort. Meier and colleagues have reported that fasting glycaemia is reduced by GLP-1 in type-2 diabetics following major surgery [14] and, in this study, pharmacological concentrations may not have reached steady state until a significant proportion of the fasting period had elapsed. The study was ceased prematurely in one patient receiving GLP-1 as the blood glucose was > 15 mmol/l and in three patients during placebo. When this occurred data were estimated using the last observation carried forward [11]. As the missing data occurred more frequently during placebo, and this approach is likely to underestimate the magnitude of the glycaemic excursion that would have eventuated, any bias would likely to be in favour of the null hypothesis. Given the outcome of studies relating to the effects of GLP-1 in ambulant type-2 patients [22] it is perhaps surprising that GLP-1 did not normalise blood glucose. This may be because the cohort comprised patients who were acutely ill - all patients required mechanical ventilation (11/11), the majority had a high APACHE II scores, approximately 50% (6/11) had kidney failure, and approximately 30% (3/11) were receiving vasoactive drugs during the study. The maximal, or near-maximal, endogenous counter-regulatory hormonal response and administration of exogenous catecholamines are likely to affect glucose tolerance adversely and, may, attenuate the glucose-lowering effect of GLP-1. Many patients admitted to the Intensive Care Unit have less severe illnesses than those studied, and the glucose-lowering effect of GLP-1 may, potentially, be greater in this group. It would be useful to be able to predict 'GLP-1 responders'. Nauck and colleagues have suggested that glucose-lowering induced by GLP-1 is diminished in hospitalised patients receiving IV nutrition that presented with acute pancreatitis, or had elevated triglyceride concentrations or higher glycated haemoglobin at baseline [13]. It is also likely that genetic variation will determine response to GLP-1 [23]. We were unable to determine which factors predicted glucose-lowering in this small sample.

The mechanism(s) underlying the glucose-lowering that occurs with GLP-1 in the critically ill are poorly defined [24] and this issue represented a secondary aim of this study. Serum insulin was increased markedly by GLP-1, but the insulinotropic effect may have been underestimated as the time between ceasing exogenous insulin and starting the study drug was only two hours which may have been insufficient for complete clearance of exogenous insulin. This time period was chosen to minimise the possibility of blood glucose concentrations > 10 mmol/l prior to commencement of the study drug. C-peptide, which is secreted in eqimolar concentrations to insulin, was unaffected. However, approximately 50% of the subjects had kidney failure, and metabolism of C-peptide is impaired to a greater degree in this group [24]. GLP-1 reduces fasting glucagon concentrations in health, ambulant type-2 diabetics, and critically ill patients with stress-hyperglycaemia [8,14,25-27] - and a reduction in glucagon was anticipated [28], but GLP-1 had no effect on Δ_glucagon _in this study. While this may suggest that hyperglucagonaemia in the critically ill diabetic patients is, relatively, resistant to suppression by GLP-1, the substantial heterogeneity of the cohort studied - in terms of pre-morbid conditions such as weight, insulin resistance and glucose control (glycated haemoglobin), as well as type and severity of acute illness - may have confounded interpretation, particularly as the sample size was relatively small. Loss of postprandial glucagon suppression is characteristic of type-2 diabetes [29]. To our knowledge this is the first report that glucagon secretion is similarly unaffected by enteral nutrient in type-2 diabetes who are critically ill. The lack of any effect of GLP-1 on 'postprandial' glucagon is, however, surprising [25,28,30]. Non-esterified fatty acids (NEFA) contribute to insulin resistance [28] and exogenous GLP-1 has been shown to have the capacity to attenuate the postprandial increase in NEFA in other groups [28,31]. In this study GLP-1 had no apparent effect on lipidaemia, but, the small intestinal nutrient infusion did not increase NEFA during placebo. Accordingly, the lack of effect on NEFA may reflect the rate of caloric delivery (1 kcal/minute) and increasing caloric load could alter this result. It should also be noted that glycaemia itself is a potent modulator of islet cell function and by not using a glycaemic clamp we may have underestimated mechanisms underlying glucose-lowering [13]. Other mediators that were not measured may also have contributed to the glycaemic effect. For example, in obese subjects the so-called 'inactive' GLP-1 metabolite (GLP-1(9-36)-NH_2_) has been reported to markedly ameliorate hepatic glucose production independent of its effects on islet cells, but concentrations of the metabolite were not measured [32].

## Conclusions

This study establishes that exogenous GLP-1 attenuates the glycaemic response to enteral nutrient in critically ill patients with type-2 diabetes. However, glycaemia was maintained at < 10 mmol/l in only approximately 50% of patients. Accordingly, the use of GLP-1 as a single agent is unlikely to be an effective treatment unless increased dose(s) have a greater effect and/or glucose-lowering is markedly greater during intragastric feeding. If this proves not to be the case future studies should, arguably, focus on critically ill patients with 'stress hyperglycaemia' rather than those with pre-existing type-2 diabetes. The effect of GLP-1 in other patient groups who are less unwell (such as high dependency care units or on discharge to general wards) also warrants evaluation.

## Key messages

• Glucagon-like peptide-1 (GLP-1) decreases blood glucose via stimulation of insulin, and suppression of glucagon secretion, as well as slowing of gastric emptying.

• The effects of GLP-1 on insulin and glucagon are glucose-dependent, therefore, the risk of hypoglycaemia with its administration is low.

• In this study exogenous GLP-1 attenuates the glycaemic response to enteral nutrient in critically ill patients with type-2 diabetes.

• However, the use of GLP-1 (at 1.2 pmol/kg/minute) maintained glycaemia at < 10 mmol/l in only approximately 50% of patients with pre-existing type-2 diabetes.

• Further study with an increased dose, administration during intragastric feeding, and/or administration with insulin warrants evaluation.

## Abbreviations

APACHE: Acute Physiology and Chronic Health Evaluation; AUC: areas under curve; BMI: body mass index; GLP-1: Glucagon-Like Peptide-1; INR: international normalised ratio; IV: Intravenous; NEFA: non-esterified fatty acid

## Competing interests

The authors declare that they have no competing interests.

## Authors' contributions

AMD was the co-contributor to study design, the acquisition, analysis and interpretation of the data and drafting the manuscript. MH was the co-contributor to study conception and participated in drafting the manuscript. MJS, AVZ and AED were responsible for data acquisition and analysis and contributed to revision of manuscript. MJC and RJLF also contributed to study conception and revision of manuscript. JMW was responsible analysis of data and contributed to revision of manuscript. All authors read and approved the final manuscript.
